# Reduced sensitivity to thyroid hormones is associated with differentiated thyroid cancer in the euthyroid thyroidectomy population

**DOI:** 10.3389/fendo.2025.1595002

**Published:** 2025-06-04

**Authors:** Huaijin Xu, Hongzhou Liu, Xiaodong Hu, Xiaomeng Jia, Zhe Xue, Anning Wang, Shaoyang Kang, Zhaohui Lyu

**Affiliations:** ^1^ School of Medicine, Nankai University, Tianjin, China; ^2^ Department of Endocrinology, First Medical Center of Chinese People’s Liberation Army (PLA) General Hospital, Beijing, China; ^3^ Department of Endocrinology, Aerospace Center Hospital, Beijing, China; ^4^ Department of Endocrinology, Sixth Medical Center of Chinese People’s Liberation Army (PLA) General Hospital, Beijing, China

**Keywords:** thyroid hormone sensitivity, differentiated thyroid cancer, thyroid hormone, thyroid homeostasis, epidemiology

## Abstract

**Background:**

The inconclusive associations between thyroid-related hormones and differentiated thyroid cancer (DTC) suggest complex pathophysiologic processes, for which thyroid hormone sensitivity may provide new insights.

**Methods:**

We retrospectively analyzed preoperative clinical data and postoperative pathological data of 9,515 euthyroid adults who underwent thyroidectomy for thyroid nodules pathologically confirmed as benign nodules or DTC. Composite thyroid parameters were calculated, including TSH index (TSHI), thyrotroph thyroxine resistance index (TT4RI), FT3/FT4 ratio (FT3/FT4) and the thyroid’s secretory capacity (SPINA-GT).

**Results:**

Increased TSHI (OR=1.34, 95%CI: 1.27-1.41) and TT4RI (OR=1.35, 95%CI: 1.28-1.42) reflecting reduced central thyroid hormone sensitivity, decreased FT3/FT4 (OR=0.81, 95%CI: 0.77-0.86) reflecting reduced peripheral thyroid hormone sensitivity, and decreased SPINA-GT (OR=0.78, 95%CI: 0.74-0.82) were associated with DTC after adjustment for confounders. The contributions of thyroid hormone sensitivity indices remained in subgroups stratified by age, sex, metabolic factors, thyroid autoimmunity status, and nodule size. A non-linear relationship between thyroid hormone sensitivity indices and probability of DTC was observed. The association of DTC with TT4RI or TSHI was stronger than with other thyroid parameters such as TSH (thyroid stimulating hormone). ROC analysis for the distinction between DTC and benign disease showed no single thyroid parameter with the coexistence of high sensitivity and specificity.

**Conclusion:**

Reduced central and peripheral sensitivity to thyroid hormones is associated with DTC in the euthyroid thyroidectomy population and provides additional information on the odds of malignancy in thyroid nodules at risk for surgery, warranting consideration of the role of sensitivity to thyroid hormones in mechanisms and prediction models for DTC.

## 1 Introduction

Thyroid cancer is one of the most common endocrine cancers with an increasing incidence in recent years (1) and differentiated thyroid cancer (DTC) accounts for more than 90% of all thyroid cancers (2). The pathogenesis of DTC has not been fully clarified. Numerous studies have investigated the association between thyroid cancer and thyroid-related hormones including thyroid stimulating hormone (TSH), triiodothyronine (T3) and thyroxine (T4), but their results were inconsistent (1, 3–6), suggesting complex pathophysiologic processes in the thyroid system. Interactions exist among T3, T4 and TSH, mediated by the hypothalamic-pituitary-thyroid (HPT) axis (7), thyroid hormone metabolism-related enzymes (8), and thyroid hormone receptors in the target organs (9). Therefore, thyroid hormone homeostasis is closely related to sensitivity to thyroid hormones. Thyroid hormone sensitivity can be assessed by thyroid composite parameters calculated from thyroid-related hormone levels. TSH index (TSHI) (10) and Thyrotroph thyroxine resistance index (TT4RI) (11), which are calculated from TSH and free thyroxine (FT4), represent the degree of pituitary inhibition by FT4 levels and thus reflect central thyroid hormone sensitivity (12). FT3/FT4 ratio (FT3/FT4) measures the peripheral deiodinase activity that mediates the conversion of FT4 to free triiodothyronine (FT3), thus reflecting peripheral thyroid hormone sensitivity. Although these calculated parameters have been used to explore the relationship with metabolic diseases (7, 12), their implications in oncology remain largely understudied.

The most recent meta-analysis (1) has indicated that thyroid cancer is associated with higher TSH and FT4, seemingly contradictory to the regulation of the negative feedback loop of the HPT axis. Apart from that, inconsistent findings from previous epidemiologic research and the lack of a pathogenic association of thyroid function with thyroid carcinogenesis in any animal or *in vitro* model (13, 14) also suggest a confusing relationship between the thyroid hormone system and thyroid cancer. Is it possible to utilize thyroid hormone sensitivity indices considering TSH and thyroid hormones simultaneously to explain this relationship? There has been an association of follicular thyroid cancer (FTC) in mouse models with some specific thyroid hormone receptor beta (THRB) mutations (15, 16) which can cause thyroid hormone resistance syndrome (17). In addition, some studies found that decreased mRNA and enzyme activity levels of deiodinases may be related to the dedifferentiation of thyroid cells towards papillary thyroid cancer (PTC) (18, 19), suggesting a potential pathophysiologic connection between thyroid hormone sensitivity and DTC.

Given that the role of thyroid hormone sensitivity in DTC remains unclear, we investigated its association with DTC in the euthyroid thyroidectomy population. We also compared the discriminative abilities of various thyroid parameters for DTC.

## 2 Materials and methods

### 2.1 Study population

**Figure 1:**
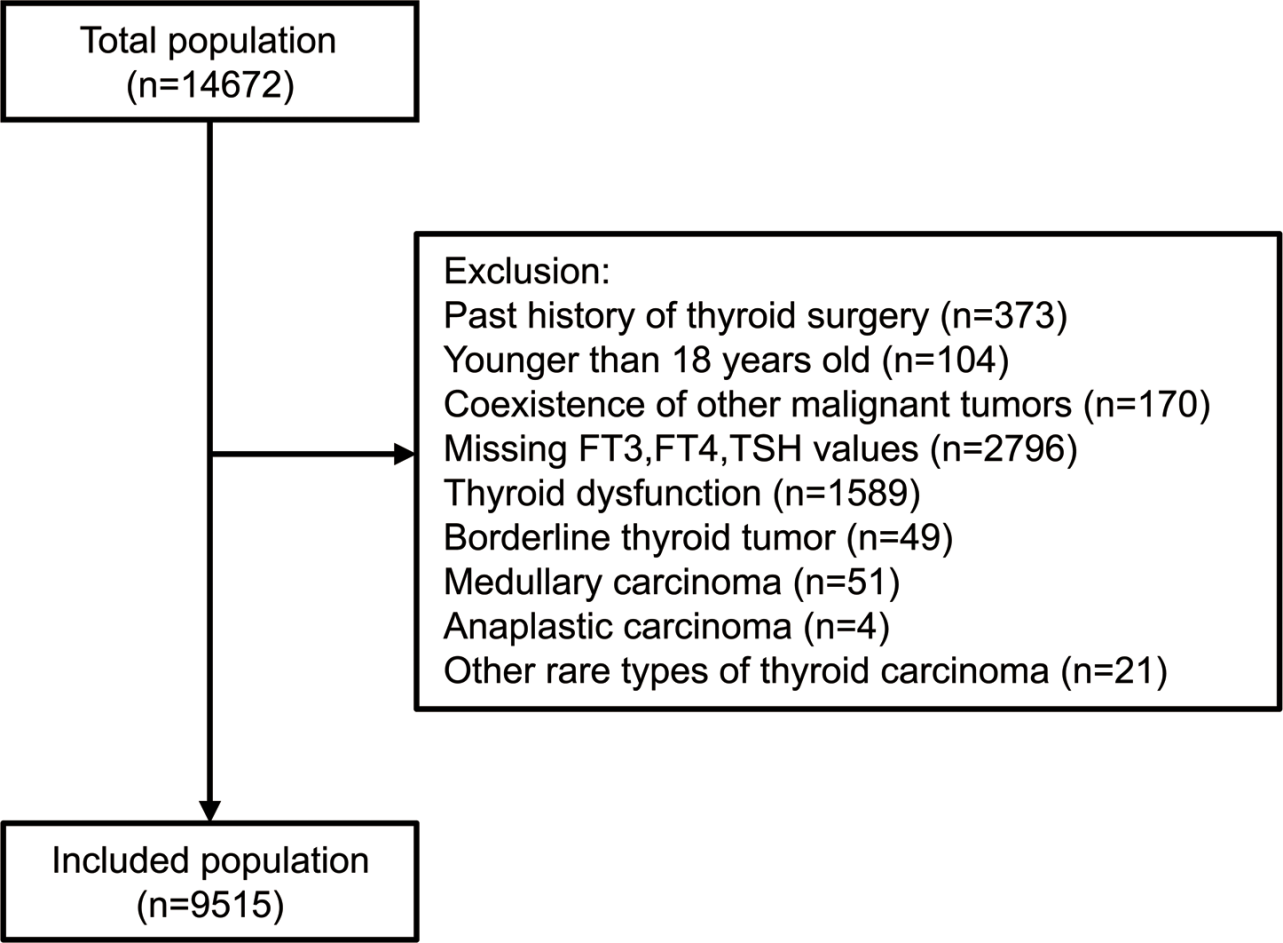
Flow chart of the selection of study subjects.

The study included all patients (n=14,672) who underwent thyroidectomy for thyroid nodules with definite pathologic diagnosis in Chinese PLA General Hospital from January 2011 to December 2020. The exclusion criteria involved past history of thyroid surgery, iodine-131 therapy or radiofrequency ablation; age<18 years; coexistence of other cancers, acute infection, liver failure, or renal failure; pregnant or breastfeeding women; missing data on FT3, FT4 or TSH; known clinical hyperthyroidism or hypothyroidism according to the electric records system or patients’ self-reports, or with FT3, FT4, TSH out of normal ranges; subjects who had taken medications which can alter thyroid function (anti-thyroid drugs, thyroid replacement drugs, amiodarone or lithium); postoperative pathology of thyroid nodules confirmed as borderline thyroid tumor (described as “non-invasive follicular thyroid neoplasm with papillary-like nuclear features, NIFTP”, “well differentiated tumor of uncertain malignant potential, WDT-UMP”, “follicular tumor of uncertain malignant potential, FT-UMP”, “hyalinizing trabecular tumor, HTT” or general descriptions like “borderline follicular tumor” in the pathology report), medullary carcinoma, anaplastic carcinoma, or other rare types of thyroid carcinoma. Finally, 9,515 individuals were included for analysis (**Figure 1**). The study was conducted following the Helsinki Declaration and approved by the Ethics Committee of Chinese PLA General Hospital with waived informed consent considering the collection of deidentified retrospective data.

### 2.2 Clinical evaluation and pathology features

The subjects received a physical examination and thyroid function test less than one week before the thyroidectomy. Height and weight were measured by trained nurses in a standardized process and body mass index (BMI) was calculated as weight(kg)/height^2^(m^2^). Serum FT3 (reference range: 2.76-6.30 pmol/L), FT4 (reference range: 10.42-24.32 pmol/L), TSH (reference range: 0.35-5.50 mU/L), thyroid peroxidase antibody (TPOAb, reference range:< 60 IU/mL) and thyroglobulin antibody (TgAb, reference range:< 60 IU/mL) were detected by the ADVIA centaur XP automated chemiluminescence analyzer (SIEMENS, USA) at the central laboratory of the hospital. Data regarding family history of thyroid cancer and the histories of related diseases were collected from the electric records system or patients’ self-reports.

The pathological type of thyroid nodule, nodule size, and the coexistence of Hashimoto thyroiditis (HT) were collected from postoperative pathology reports. According to the standard diagnostic workflow in the Pathology Department, after a pathologist provided an initial report of pathological findings, a senior pathologist conducted a secondary review. For cases with discrepancies, consensus was reached through collective discussion. Nodule size was defined as the maximum pathologic diameter of benign nodules for patients with benign thyroid disease and malignant nodules for patients with DTC.

### 2.3 Calculation of the thyroid composite indices

1. TSH Index (TSHI) = Ln TSH(mU/L) + 0.1345 × FT_4_(pmol/L) (10).
2. Thyrotroph Thyroxine Resistance Index (TT4RI) = FT_4_(pmol/L) × TSH(mU/L) (11).
3. FT3/FT4 ratio (FT3/FT4) = FT_3_(pmol/L)/FT_4_(pmol/L).
4. The Thyroid’s Secretory Capacity (SPINA-GT) = β_T_ (D_T_ + [TSH(mU/L)]) (1+K_41_[TBG]+K_42_[TBPA])[FT_4_(pmol/L)]/(α_T_[TSH(mU/L)]) (20).

where *β_T_* (clearance exponent for T4) = 1.1 × 10^−6^/s, *D_T_* (EC_50_ for TSH) = 2.75 mU/L, K_41_ (dissociation constant of T4 at thyroxine-binding globulin) = 2.0 × 10^10^ L/mol, TBG (standard concentration of thyroxine-binding globulin) = 300 nmol/L, *K*_42_ (dissociation constant of T4 at transthyretin) = 2.0 × 10^8^ L/mol, TBPA (standard transthyretin concentration) = 4.5 *μ*mol/L, and *α*_T_ (dilution factor for thyroxine) = 0.1/L (20). SPINA-GT is defined as the maximum amount of T4 that the thyroid can release in a given time unit under stimulated conditions, as proposed and validated by Dietrich et al. (20). Here we calculated SPINA-GT using R software with the “SPINA” package (20, 21). No winsorizing or trimming was applied to thyroid parameters in consideration that euthyroid status inherently constrained extreme deviations.

Higher TSHI or TT4RI indicates lower central thyroid hormone sensitivity, and higher FT3/FT4 indicates higher peripheral thyroid hormone sensitivity. The value of SPINA-GT reflects the maximum secretory capacity of the thyroid gland (20).

### 2.4 Statistical analysis

Continuous variables were expressed as mean ± standard deviation (SD) for normally distributed data and median (interquartile range) for skewed distributed data. Categorical variables were expressed as numbers (percentages). Independent samples t-test and Mann-Whitney U test were used for between-group comparisons of normally and skewed distributed variables, respectively. The chi-square test was used for categorical variables. TSH, TT4RI, FT3/FT4, and SPINA-GT were natural log (ln) transformed in further analyses given their skewed distributions. Different logistic regression models were constructed to estimate odds ratios (OR) with 95% confidence intervals (95%CI) for DTC across quartiles or per SD increase of thyroid parameters. Quartile-based analysis focused on evaluating categorical trends, while SD-change analysis quantified associations across continuous distributions. These approaches analyzed the data from distinct analytical dimensions. The crude model was without adjustment; Model 1 was adjusted for age and sex; Model 2 was further adjusted for BMI, family history of thyroid cancer and Hashimoto thyroiditis confirmed by pathology. The covariates included in the models were potential confounding and/or mediating factors for the relationship between thyroid function and DTC. As the proportion of missing data for each covariate was less than 5% (**Supplementary Table S1**), missing data were not processed.

Stratified logistic regression analyses were performed in subgroups stratified by age 55 (age for the TNM staging of DTC (22)), sex, BMI (Chinese BMI classification (23): BMI<24, underweight and normal weight; 24≤BMI<28, overweight; BMI≥28, obese), diabetes, hypertension, Hashimoto thyroiditis, the status of thyroid autoantibodies, nodule size and surgery periods, with their interactions tested. Generalized additive model and smoothed curve fitting were used to provide more detailed information about the relationship between thyroid hormone sensitivity indices (not ln-transformed for clearer and more intuitive visualization) and the probability of DTC. When significant nonlinearity was detected via likelihood ratio testing, the inflection point was calculated using a recursive algorithm and the threshold effects were analyzed using segmented regression models. 95% CIs for inflection points were estimated using bootstrap resampling with 1,000 iterations. The degrees of association between DTC and various thyroid parameters were assessed by comparing the standardized regression coefficient β of each thyroid parameter in the multivariable-adjusted model and receiver operating characteristic (ROC) curve analysis was conducted. All analyses were performed with SPSS version 26 and R software version 4.3.1. P values<0.05 (2-sided) were considered significant statistically.

## 3 Results

### 3.1 Characteristics of the study population

The study included 9,515 individuals, of whom 7,447 suffered from DTC. The average age of the overall population was 45.47 ± 11.38 years, and 28.9% were male. Compared to subjects with benign disease, subjects with DTC exhibited younger age, higher BMI, TgAb and TPOAb levels, smaller nodule size, and higher prevalences of Hashimoto thyroiditis confirmed by pathology and family history of thyroid cancer (all P<0.05), with no statistical difference in sex distribution, history of external neck irradiation, hypertension, or diabetes. In terms of thyroid parameters, FT4, TSH, TSHI, and TT4RI were higher, while FT3, FT3/FT4, and SPINA-GT were lower in subjects with DTC (all P<0.001) (**Table 1**). Vice versa, the proportions of DTC increased across quartiles of TSH, TSHI, and TT4RI but decreased with rising quartiles of FT3, FT3/FT4, and SPINA-GT (all P<0.001). Differences existed in the proportions of pathology for FT4 quartile groups (P<0.001) but not concentration-dependently (**Supplementary Figure S1**).

**Table 1:** Characteristics of euthyroid subjects with benign disease or differentiated thyroid cancer.

	Total	Benign disease	Differentiated thyroid cancer	P value
N	9515	2068	7447	
Age (years)	45.47 ± 11.38	50.04 ± 11.87	44.20 ± 10.91	<0.001
Male, n (%)	2752 (28.9)	573 (27.7)	2179 (29.3)	0.168
BMI (kg/m^2^)	24.85 ± 3.58	24.55 ± 3.47	24.93 ± 3.60	<0.001
FT3 (pmol/L)	4.70 ± 0.52	4.75 ± 0.50	4.69 ± 0.52	<0.001
FT4 (pmol/L)	15.16 ± 2.15	15.02 ± 2.22	15.20 ± 2.13	<0.001
TSH (mU/L)	1.89 (1.27, 2.74)	1.66 (1.06, 2.49)	1.96 (1.34, 2.81)	<0.001
TSHI	2.65 ± 0.59	2.50 ± 0.64	2.69 ± 0.56	<0.001
TT4RI	28.62 (19.37, 41.11)	24.88 (15.87, 37.12)	29.72 (20.24, 42.04)	<0.001
FT3/FT4	0.31 (0.28, 0.34)	0.32 (0.29, 0.35)	0.31 (0.28, 0.34)	<0.001
SPINA-GT (pmol/s)	2.80 (2.22, 3.69)	3.00 (2.30, 4.19)	2.76 (2.20, 3.58)	<0.001
TgAb (IU/ml)	16.50 (15.00, 42.65)	15.00 (15.00, 27.10)	17.00 (15.00, 53.53)	<0.001
TPOAb (IU/ml)	29.40 (27.00, 44.10)	28.20 (27.00, 40.60)	29.60 (27.00, 45.10)	<0.001
Hashimoto thyroiditis confirmed by pathology, n (%)	1329 (14.0)	112 (5.4)	1217 (16.4)	<0.001
Nodule size (cm)	1.00 (0.60, 2.00)	3.00 (1.50, 4.00)	1.00 (0.60, 1.50)	<0.001
History of external neck irradiation, n (%)	22 (0.2)	6 (0.3)	16 (0.2)	0.528
Family history of thyroid cancer, n (%)	105 (1.1)	10 (0.5)	95 (1.3)	0.002
Hypertension, n (%)	2963 (31.1)	644 (31.1)	2319 (31.1)	0.999
Diabetes, n (%)	564 (5.9)	113 (5.5)	451 (6.1)	0.313

Data are expressed as mean ± standard deviation for normally distributed variables, median (interquartile range) for skewed distributed variables, and numbers (percentages) for categorical variables.

BMI, body mass index; FT3, free triiodothyronine; FT4, free thyroxine; TSH, thyroid stimulating hormone; TSHI, TSH index; TT4RI, thyrotroph thyroxine resistance index; FT3/FT4, FT3/FT4 ratio; SPINA-GT, the thyroid’s secretory capacity; TgAb, serum thyroglobulin antibody; TPOAb, serum thyroid peroxidase antibody.

### 3.2 The association between thyroid parameters and DTC in total subjects

Reduced FT3, FT3/FT4, or SPINA-GT was associated with DTC: After adjustment for age, sex, BMI, family history of thyroid cancer and Hashimoto thyroiditis confirmed by pathology, for each SD change in FT3, FT3/FT4, and SPINA-GT, the ORs (95% CI) for DTC were 0.79 (0.75-0.83), 0.81 (0.77-0.86), and 0.78 (0.74-0.82), respectively (**Table 2**). Analysis comparing extreme quartiles (Q1 vs. Q4) showed similar associations (**Supplementary Table S2**), with subjects in the lowest quartiles (Q1) of FT3, FT3/FT4, and SPINA-GT demonstrating a significantly higher risk of DTC compared to those in Q4. Increased FT4 was associated with DTC in the crude model, which was no longer significant after adjustment for confounders (**Table 2**). However, subjects with the highest quartile of FT4 still had a higher prevalence of DTC than those with the lowest quartile in all models (all P values < 0.05) (**Supplementary Table S2**). Increased TSH, TSHI or TT4RI was associated with DTC: After adjustment for confounders, for each SD change in TSH, TSHI, and TT4RI, the ORs (95% CI) for DTC were 1.33 (1.26-1.40), 1.34 (1.27-1.41), and 1.35 (1.28-1.42), respectively (**Table 2**). Quartile analysis showed analogous results (**Supplementary Table S2**), with subjects in the highest quartiles (Q4) of TSH, TSHI, and TT4RI demonstrating a significantly higher risk of DTC compared to those in Q1.

**Table 2:** Association between thyroid parameters (per SD change) and differentiated thyroid cancer in total subjects.

	Crude		Model 1		Model 2	
	OR (95%CI)	P value	OR (95%CI)	P value	OR (95%CI)	P value
FT3	0.88 (0.84-0.93)	<0.001	0.78 (0.74-0.83)	<0.001	0.79 (0.75-0.83)	<0.001
FT4	1.09 (1.04-1.15)	0.001	1.05 (1.00-1.11)	0.068	1.05 (1.00-1.11)	0.062
TSH	1.36 (1.30-1.43)	<0.001	1.35 (1.29-1.42)	<0.001	1.33 (1.26-1.40)	<0.001
TSHI	1.40 (1.33-1.47)	<0.001	1.36 (1.29-1.43)	<0.001	1.34 (1.27-1.41)	<0.001
TT4RI	1.39 (1.33-1.46)	<0.001	1.37 (1.30-1.44)	<0.001	1.35 (1.28-1.42)	<0.001
FT3/FT4	0.83 (0.79-0.87)	<0.001	0.81 (0.77-0.85)	<0.001	0.81 (0.77-0.86)	<0.001
SPINA-GT	0.78 (0.74-0.82)	<0.001	0.77 (0.73-0.81)	<0.001	0.78 (0.74-0.82)	<0.001

The ORs (95% CI) of continuous thyroid parameters (per SD change) for differentiated thyroid cancer in different logistic regression models are shown.

Model 1: Adjusted for age and sex;

Model 2: Adjusted for age, sex, BMI, family history of thyroid cancer and Hashimoto thyroiditis confirmed by pathology.

FT3, free triiodothyronine; FT4, free thyroxine; TSH, thyroid stimulating hormone; TSHI, TSH index; TT4RI, thyrotroph thyroxine resistance index; FT3/FT4, FT3/FT4 ratio; SPINA-GT, the thyroid’s secretory capacity.

### 3.3 The association between thyroid parameters and DTC in subgroups

The ORs (95%CI) of per SD increase in thyroid parameters for DTC were calculated in subgroups after adjustment for potential confounders (**Table 3**). In subjects stratified by age, sex, BMI, diabetes or hypertension, TSHI and TT4RI were positively associated with DTC, while FT3/FT4 and SPINA-GT were negatively associated with DTC (all P values <0.05). The association of DTC with TSHI, TT4RI or SPINA-GT was stronger in females than in males (*P* for interaction <0.05). In addition, there was a statistically significant interaction between FT3/FT4 and BMI with DTC (*P* for interaction = 0.038).

**Table 3:** Association between thyroid parameters (per SD change) and differentiated thyroid cancer in subgroups.

	TSHI	TT4RI	FT3/FT4	SPINA-GT
Age ^a^				
<55 years old (N=7392)	**1.29 (1.21-1.38)**	**1.31 (1.23-1.40)**	**0.83 (0.78-0.89)**	**0.78 (0.74-0.84)**
≥55 years old (N=2102)	**1.42 (1.30-1.55)**	**1.41 (1.29-1.54)**	**0.77 (0.70-0.84)**	**0.77 (0.70-0.84)**
*P* for interaction	0.071	0.124	0.086	0.400
Sex ^b^				
Male (N=2745)	**1.20 (1.08-1.32)**	**1.19 (1.08-1.32)**	**0.85 (0.77-0.94)**	**0.87 (0.79-0.97)**
Female (N=6749)	**1.39 (1.31-1.72)**	**1.40 (1.32-1.49)**	**0.79 (0.75-0.84)**	**0.75 (0.70-0.79)**
*P* for interaction	**0.016**	**0.008**	0.198	**0.003**
BMI ^a^				
BMI<24 (N=4034)	**1.37 (1.27-1.48)**	**1.35 (1.25-1.46)**	**0.75 (0.70-0.82)**	**0.81 (0.75-0.88)**
24≤BMI<28 (N=3750)	**1.31 (1.21-1.42)**	**1.35 (1.24-1.46)**	**0.87 (0.80-0.94)**	**0.74 (0.68-0.80)**
BMI≥28 (N=1710)	**1.31 (1.15-1.49)**	**1.33 (1.17-1.51)**	**0.79 (0.69-0.89)**	**0.80 (0.70-0.91)**
*P* for interaction	0.750	0.962	**0.038**	0.249
Histories of diabetes ^a^				
Yes (N=563)	**1.62 (1.29-2.03)**	**1.63 (1.30-2.05)**	**0.70 (0.56-0.88)**	**0.69 (0.56-0.85)**
No (N=8931)	**1.33 (1.26-1.40)**	**1.33 (1.27-1.41)**	**0.82 (0.78-0.86)**	**0.78 (0.74-0.83)**
*P* for interaction	0.131	0.145	0.266	0.464
Histories of hypertension ^a^				
Yes (N=2962)	**1.39 (1.27-1.52)**	**1.41 (1.29-1.54)**	**0.79 (0.72-0.87)**	**0.74 (0.68-0.81)**
No (N=6532)	**1.31 (1.23-1.39)**	**1.31 (1.23-1.40)**	**0.82 (0.77-0.87)**	**0.80 (0.75-0.85)**
*P* for interaction	0.230	0.169	0.460	0.128
Hashimoto thyroiditis confirmed by pathology ^c^				
Yes (N=1329)	**1.21 (1.00-1.47)**	1.16 (0.96-1.41)	**0.67 (0.55-0.83)**	0.97 (0.80-1.19)
No (N=8165)	**1.35 (1.28-1.42)**	**1.36 (1.29-1.43)**	**0.82 (0.77-0.86)**	**0.77 (0.73-0.81)**
*P* for interaction	0.299	0.128	**0.044**	**0.032**
TPOAb status^c^				
Positive (N=1517)	**1.51 (1.29-1.76)**	**1.49 (1.28-1.73)**	**0.78 (0.66-0.91)**	**0.75 (0.65-0.88)**
Negative (N=7141)	**1.32 (1.24-1.40)**	**1.33 (1.25-1.41)**	**0.81 (0.76-0.86)**	**0.78 (0.74-0.83)**
Unknown (N=850)	**1.41 (1.22-1.62)**	**1.43 (1.24-1.65)**	**0.85 (0.74-0.96)**	**0.74 (0.64-0.85)**
*P* for interaction	0.211	0.239	0.719	0.601
TgAb status ^c^				
Positive (N=1912)	**1.31 (1.14-1.52)**	**1.30 (1.13-1.50)**	**0.80 (0.69-0.94)**	**0.83 (0.72-0.95)**
Negative (N=7039)	**1.35 (1.27-1.43)**	**1.36 (1.28-1.44)**	**0.80 (0.75-0.85)**	**0.77 (0.73-0.82)**
Unknown (N=557)	**1.24 (1.05-1.46)**	**1.27 (1.08-1.50)**	**0.85 (0.74-0.99)**	**0.80 (0.68-0.94)**
*P* for interaction	0.605	0.748	0.751	0.784
Nodule size ^a^				
≤1cm (N=3976)	**1.19 (1.04-1.37)**	**1.18 (1.03-1.35)**	**0.79 (0.68-0.91)**	0.89 (0.77-1.03)
>1cm (N=3665)	**1.35 (1.26-1.45)**	**1.37 (1.28-1.48)**	**0.82 (0.76-0.88)**	**0.75 (0.70-0.81)**
Unknown (N=1853)	**1.35 (1.22-1.50)**	**1.34 (1.21-1.49)**	**0.80 (0.72-0.89)**	**0.81 (0.73-0.89)**
*P* for interaction	0.233	0.150	0.759	0.142
Surgery period ^a^				
**2011-2015 (N=4324)**	**1.33 (1.24-1.42)**	**1.36 (1.27-1.45)**	**0.90 (0.84-0.96)**	**0.75 (0.70-0.80)**
**2016-2020 (N=5170)**	**1.39 (1.27-1.52)**	**1.35 (1.24-1.47)**	**0.69 (0.63-0.76)**	**0.83 (0.77-0.91)**
***P* for interaction**	0.396	0.967	**<0.001**	0.085

The ORs (95%CI) of per SD increase in thyroid parameters for differentiated thyroid cancer are shown. Bold indicates P value < 0.05.

a Adjusted for age, sex, BMI, family history of thyroid cancer, and Hashimoto thyroiditis confirmed by pathology;

b Adjusted for age, BMI, family history of thyroid cancer, and Hashimoto thyroiditis confirmed by pathology;

c Adjusted for age, sex, BMI, and family history of thyroid cancer.

TSHI, TSH index; TT4RI, thyrotroph thyroxine resistance index; FT3/FT4, FT3/FT4 ratio; SPINA-GT, the thyroid’s secretory capacity; TPOAb, serum thyroid peroxidase antibody; TgAb, serum thyroglobulin antibody.

In subjects with HT confirmed by pathology, the association of TT4RI or SPINA-GT with DTC was no longer significant. Additionally, there was a statistically significant interaction between FT3/FT4 and HT with DTC, similarly for SPINA-GT. However, in both thyroid autoantibody-positive and negative individuals, all thyroid hormone sensitivity indices and SPINA-GT were significantly associated with DTC. No statistical heterogeneity in this association was observed in subgroups of different statuses of autoantibodies (all *P* for interaction >0.05). The contribution of thyroid hormone sensitivity indices remained in subgroups of nodule size (all *P* for interaction >0.05). And the association of SPINA-GT with DTC was no longer statistically significant in subjects with nodule size ≤1cm. Subgroup analysis between the surgical periods of 2011–2015 and 2016–2020 demonstrated results consistent with those in the total population.

### 3.4 Non-linear relationship between thyroid hormone sensitivity indices and the probability of DTC

**Figure 2:**
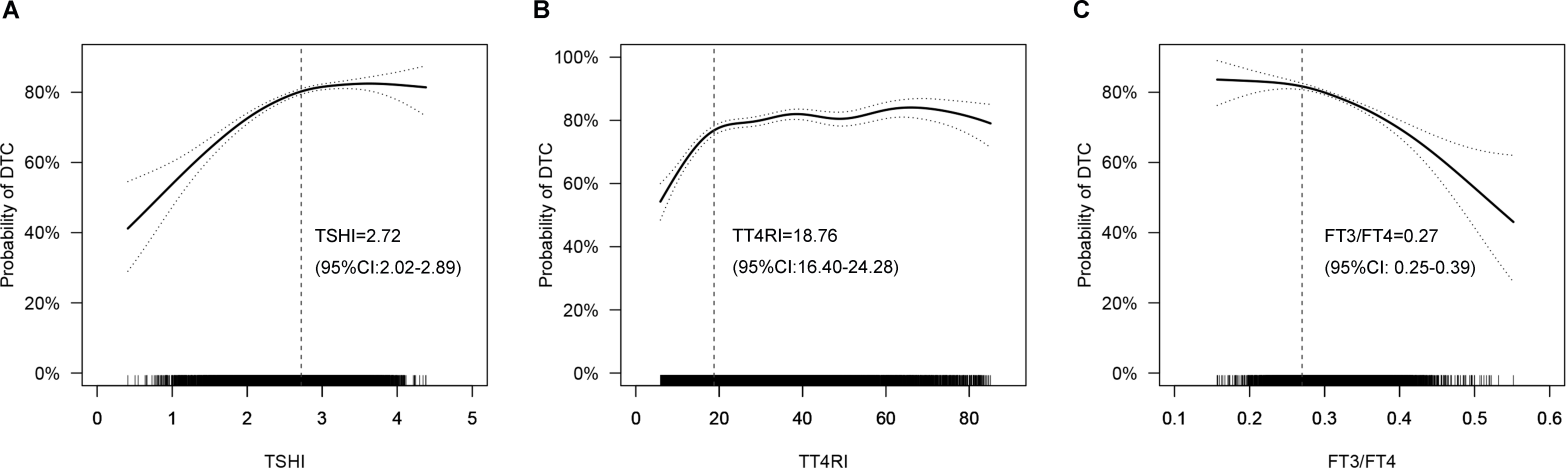
Smoothed curve fitting: Non-linear relationships between thyroid hormone sensitivity indices and the probability of DTC (**A**: TSHI, **B**: TT4RI, **C**: FT3/FT4). The solid line represents the fitted curve, while the dotted line represents the 95% confidence interval. Age, sex, BMI, family history of thyroid cancer and hashimoto thyroiditis were adjusted. DTC, differentiated thyroid cancer; TSHI, TSH index; TT4RI, thyrotropin thyroxine resistance index; FT3/FT4: FT3/FT4 ratio.

Adjusted smoothed curves suggested a non-linear relationship between thyroid hormone sensitivity indices and the probability of DTC (**Figure 2**). Threshold effect analysis showed that the inflection points were 2.72 (95% CI: 2.02-2.89), 18.76 (95% CI: 16.40-24.28) and 0.27 (95% CI: 0.25-0.39) for TSHI, TT4RI and FT3/FT4, respectively. The probability of DTC increased with TSHI up to the inflection point (2.72) (OR 2.125, 95% CI 1.850-2.441, P<0.001). When TSHI was >2.72, the relationship between TSHI and the probability of DTC was not significant (OR 1.024, 95% CI 0.834-1.264, P= 0.820). The probability of DTC increased with a more prominent trend when TT4RI was below the inflection point (18.76) (OR 1.087, 95% CI 1.067-1.108, P<0.001). No significant association was found when FT3/FT4 was <0.27 (OR 10.213, 95% CI 0.072-1237.818, P=0.350); however, the probability of DTC decreased with FT3/FT4 when FT3/FT4 was >0.27 (OR 0.003, 95% CI 0.001-0.011, P<0.001). These results suggested a strong and significant association of higher central and peripheral thyroid hormone sensitivity with lower risk of DTC in the thyroidectomy population.

### 3.5 Comparison of the roles of thyroid parameters in DTC

The association of DTC with central thyroid hormone sensitivity indices (TT4RI and TSHI) was stronger than with other thyroid parameters such as TSH (**Table 4**). Based on the ROC curves for the distinction between DTC and benign disease (**Supplementary Figures S2**, **S3**), among various thyroid parameters, the highest sensitivity was 74.43% in TSH, and the highest specificity was 82.40% in SPINA-GT. The area under the curve (AUC) for TSHI or TT4RI was higher than that for TSH; nevertheless, there was no single thyroid parameter with the coexistence of high sensitivity and specificity (**Table 5**).

**Table 4:** Degrees of association between thyroid parameters and differentiated thyroid cancer.

	B	β	OR (95%CI)	*P*
FT3	-0.2472	-0.1360	0.789 (0.746-0.834)	<0.001
FT4	0.0520	0.0282	1.052 (0.998-1.109)	0.062
TSH	0.2822	0.1541	1.328 (1.262-1.397)	<0.001
TSHI	0.2893	0.1585	1.336 (1.270-1.406)	<0.001
TT4RI	0.2952	0.1616	1.345 (1.278-1.415)	<0.001
FT3/FT4	-0.2160	-0.1178	0.813 (0.772-0.857)	<0.001
SPINA-GT	-0.2459	-0.1334	0.781 (0.742-0.821)	<0.001

The non-standardized regression coefficient (B), standardized regression coefficient (β), and ORs (95%CI) are shown.

The degrees of association between differentiated thyroid cancer and various thyroid parameters were assessed by comparing β of each thyroid parameter in the logistic regression model adjusted for age, sex, BMI, family history of thyroid cancer, and Hashimoto thyroiditis confirmed by pathology.

FT3, free triiodothyronine; FT4, free thyroxine; TSH, thyroid stimulating hormone; TSHI, TSH index; TT4RI, thyrotroph thyroxine resistance index; FT3/FT4, FT3/FT4 ratio; SPINA-GT, the thyroid’s secretory capacity.

**Table 5:** Performance of thyroid parameters for distinction between DTC and benign disease.

	AUC (95%CI)	Cut-off value	Sensitivity	Specificity	Youden index
For prediction of thyroid cancer					
TSH	0.579(0.565,0.594)	1.37mIU/L	74.43%	37.86%	0.123
TSHI	0.590(0.575,0.604)	2.60	58.40%	55.80%	0.142
TT4RI	0.587(0.573,0.601)	23.76	65.61%	47.39%	0.130
For prediction of benign disease					
FT3	0.540(0.526,0.554)	4.64pmol/L	59.04%	47.94%	0.070
FT3/FT4	0.555(0.540,0.569)	0.31	60.83%	47.79%	0.086
SPINA-GT	0.562(0.547,0.576)	4.01pmol/s	28.30%	82.40%	0.107

The area under the curve (AUC), cut-off value, sensitivity, specificity, and Youden index of each thyroid parameter for the distinction between DTC and benign disease are listed based on the ROC curve analysis.

TSH, thyroid stimulating hormone; TSHI, TSH index; TT4RI, thyrotroph thyroxine resistance index; FT3, free triiodothyronine; FT3/FT4, FT3/FT4 ratio; SPINA-GT, the thyroid’s secretory capacity.

## 4 Discussion

In this study, we found that increased TSHI and TT4RI, while decreased FT3/FT4 and SPINA-GT, were associated with DTC in the euthyroid thyroidectomy population. Reduced central and peripheral thyroid hormone sensitivity could be a risk factor and a supplementary marker for DTC.

As mentioned earlier, the inconclusive associations between thyroid-related hormones and thyroid cancer reported by previous studies suggest a complex relationship between thyroid cancer and the thyroid hormone system, for which thyroid hormone sensitivity may provide new insights. Although a potential pathophysiologic connection exists, we retrieved only two related studies on PTC and no clinical studies on FTC or DTC: The cross-sectional study involving 1,998 patients undergoing thyroidectomy by Sun et al. (24) found that TSHI and TT4RI were positively associated, while FT3/FT4 was negatively associated with PTC. The other cross-sectional study including 1,594 patients by Muhanhali et al. (25) also showed a positive association of PTC with TSHI and TT4RI, consistent with our study.

In our study, we investigated the association between thyroid hormone sensitivity indices and DTC in a large sample size. Furthermore, we found that the associations between thyroid hormone sensitivity and DTC were stronger in females than in males. Our subgroup analyses for nodules ≤1 cm and >1 cm indicated the inverse association of thyroid hormone sensitivity with both differentiated thyroid microcarcinoma and non-microcarcinoma.

In addition, we investigated the relationship between DTC and SPINA-GT for the first time. SPINA-GT, a calculated parameter derived from mathematical modeling of pituitary-thyroid feedback, estimates the maximum secretion rate of T4 during thyroid stimulation, reflecting thyroid homeostasis (20). SPINA-GT has been validated and applied in numerous studies involving different ethnicities and thyroid function statuses (26–31). It can discriminate primary functional thyroid disorders from euthyroidism. Unlike conventional indicators like TSH, SPINA-GT is unaffected by hypothalamic-pituitary dysfunction (20), enhancing its clinical applicability. Retrospective data showed that SPINA-GT was higher in patients with diffuse and nodular goiter within the euthyroid range but lower in patients with autoimmune thyroiditis compared to controls (20), which may explain the higher SPINA-GT in the benign disease group (approximately 69% of benign diseases in our study were nodular goiters according to **Supplementary Table S3**) and why the association between DTC and SPINA-GT was affected by HT in our study.

To identify the most robust biomarkers, we compared the roles of various thyroid parameters in DTC for the first time. Numerous epidemiologic studies have suggested that TSH is predictive of thyroid cancer and have proposed diagnostic models containing TSH (32). However, the sensitivity and specificity of TSH are not satisfying in studies focused on TSH (33). In our thyroidectomy population, the associations of DTC with central thyroid hormone sensitivity indices were stronger than with TSH. This finding encouraged further ROC analysis for the distinction between DTC and benign disease: TSH had a higher sensitivity but a lower specificity compared with other thyroid parameters. SPINA-GT had the highest specificity. Nevertheless, just like TSH, due to the absence of both high sensitivity and specificity, thyroid hormone sensitivity indices in isolation are not reliable diagnostic markers and can only provide additional information for the odds of malignancy in nodules at risk for surgery, which may aid in clinical decision-making when combined with ultrasound features or existing diagnostic frameworks.

The mechanisms of the inverse association of DTC with thyroid hormone sensitivity are unclear given the lack of relevant research. There are several speculations as follows: (1) Reduced expression and activity of peripheral deiodinase: Type I (D1) and Type II (D2) iodothyronine deiodinases convert T4 to T3, closely related to thyroid hormone sensitivity. Some studies (18, 19) found that mRNA and enzyme activity levels of D1 and D2 deiodinases were significantly decreased in PTC tissues compared to controls, probably attributable to the dedifferentiation of thyroid cells towards PTC. (2) Mutations of thyroid hormone receptors (TR): Sequencing analysis (34) showed a high frequency of TR mutations in PTCs. Moreover, thyroid hormone resistance syndrome is mainly caused by the mutated THRB gene (24). Suzuki et al. (16) found that mice harboring a carboxyl-terminal 14 amino acid frame-shift mutation in THRB gene (TRβPV mouse) developed FTC spontaneously, and subsequent studies (15) indicated that TRβPV functioned as an oncogene in thyroid cancer via nucleus-initiated transcription as well as nongenomic signaling pathways. (3) Metabolic factors: The thyroid hormone system can regulate carbohydrate and lipid metabolism. Recent cross-sectional surveys have found an association of impaired thyroid hormone sensitivity with metabolic diseases (7, 12, 35). Insulin resistance is very common in the population with obesity, diabetes and metabolic syndrome, which may increase the risk of thyroid cancer via the activation of the IGF pathway and the insulin pathway (36). And hyperinsulinemia can induce mitogenic and anti-apoptotic effects in cells (37). Increased leptin secretion in the obese state activates various signaling pathways to modulate the growth and proliferation of thyroid carcinoma cells (36). Meanwhile, chronic inflammation in adipose tissues increases the secretion of cytokines including IL-6 and TNF, which may contribute to cancer development (38). Our subgroup analyses suggested the interaction between FT3/FT4 and BMI with DTC but didn’t support the role of diabetes or hypertension in the association between thyroid hormone sensitivity and DTC. Our study didn’t involve other metabolic factors such as abdominal obesity and serum lipids, requiring further studies. (4) Inflammation and thyroid autoimmunity: Hashimoto thyroiditis (HT), an autoimmune thyroid disease characterized by lymphocytic infiltration reflecting inflammation, is related to thyroid cancer in many studies (39). HT can lead to thyroid dysfunction and Gavin et al. (40) proposed the hypothesis that resistance to thyroid hormone can lead to HT. We also found that central and peripheral thyroid hormone sensitivity was lower in individuals with HT than those without HT (**Supplementary Table S4**). Therefore, thyroid autoimmune disease may be a mediator of the association between thyroid hormone sensitivity and DTC. Our subgroup analysis suggested that the associations between DTC and certain thyroid hormone sensitivity indices were affected by pathologically confirmed HT but not the status of thyroid autoantibodies. Further basic and clinical studies are necessary regarding the role of inflammation and immune microenvironment in this relationship. In addition, the stronger association of DTC with central thyroid hormone sensitivity indices (TT4RI and TSHI) suggests that alterations in central thyroid hormone regulation may play a more prominent role in DTC, warranting further studies to elucidate the underlying mechanisms.

Several limitations exist in our study. First, data containing thyroid autoantibodies or nodule size were absent in a small proportion of cases, so we analyzed the missing data separately, yet we couldn’t avoid the reduction in certain subgroup counts, which may yield unstable results for subgroup analysis. Second, additional metabolic factors such as insulin resistance, lipid profiles, and smoking status which may confound the association between thyroid hormone sensitivity and DTC were not included in our analysis. Third, since the study population consisted of individuals undergoing thyroidectomy for nodules and the indications for surgery resulted in a higher proportion of DTC than benign disease, the results only apply to the population with nodules at risk for surgery, and further research in the general population is warranted. Fourth, the study was single-center, but patients at our institution were from all over the north of China. Finally, only associations rather than causality can be established due to the cross-sectional design.

In conclusion, reduced central and peripheral sensitivity to thyroid hormones is a risk factor for DTC in the euthyroid thyroidectomy population and a supplementary marker for predicting the odds of malignancy in thyroid nodules at risk for surgery, warranting consideration of the role of thyroid hormone sensitivity in future studies on mechanisms and diagnostic models for DTC.

## Data Availability

The raw data supporting the conclusions of this article will be made available by the authors, without undue reservation.
