# Supplementary material for: Reduced sensitivity to thyroid hormones is associated with differentiated thyroid cancer in the euthyroid thyroidectomy population
Source: Front Endocrinol (Lausanne). 2025 Jun 4;16:1595002. doi: 10.3389/fendo.2025.1595002 (PMC12173894; doi:10.3389/fendo.2025.1595002)
Supplement: Supplementary file 1 [file DataSheet1.docx]

Supplementary Material

Reduced sensitivity to thyroid hormones is associated with differentiated thyroid cancer in the euthyroid thyroidectomy population

**Huaijin Xu^1,2^, Hongzhou Liu^3^, Xiaodong Hu^4^, Xiaomeng Jia^2^, Zhe Xue^2^, Anning Wang^2^, Shaoyang Kang^2^, Zhaohui Lyu^1,2*^**

*** Correspondence:** Zhaohui Lyu: [metabolism301@126.com](mailto:metabolism301@126.com)

**Supplementary Figures**


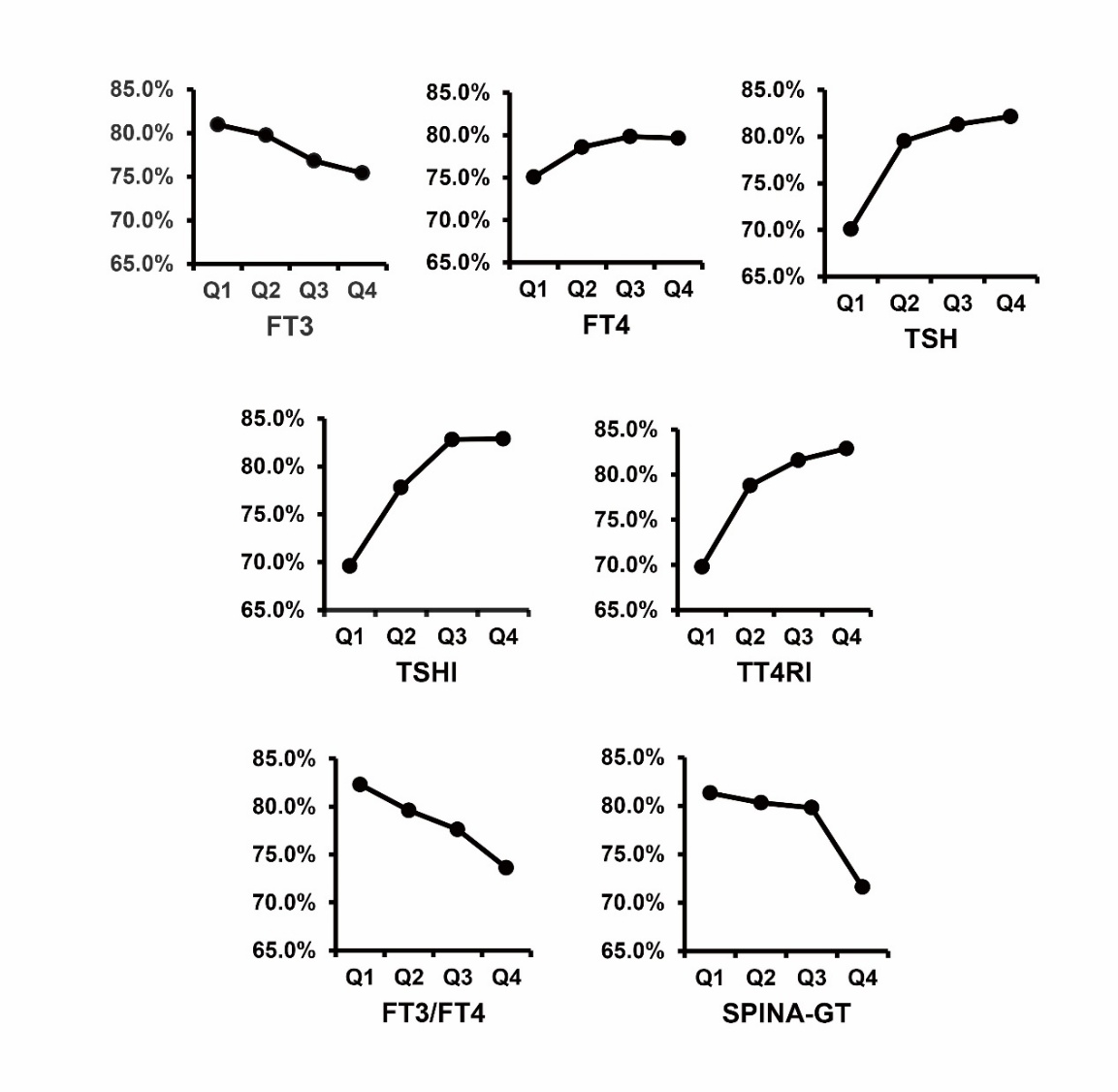


**Supplementary Figure S1.** Proportions of differentiated thyroid cancer in quartile groups of various thyroid parameters. FT3: free triiodothyronine; FT4: free thyroxine;TSH: thyroid stimulating hormone; TSHI: TSH index; TT4RI: thyrotroph T4 resistance index; FT3/FT4: FT3/FT4 ratio; SPINA-GT: the thyroid’s secretory capacity.


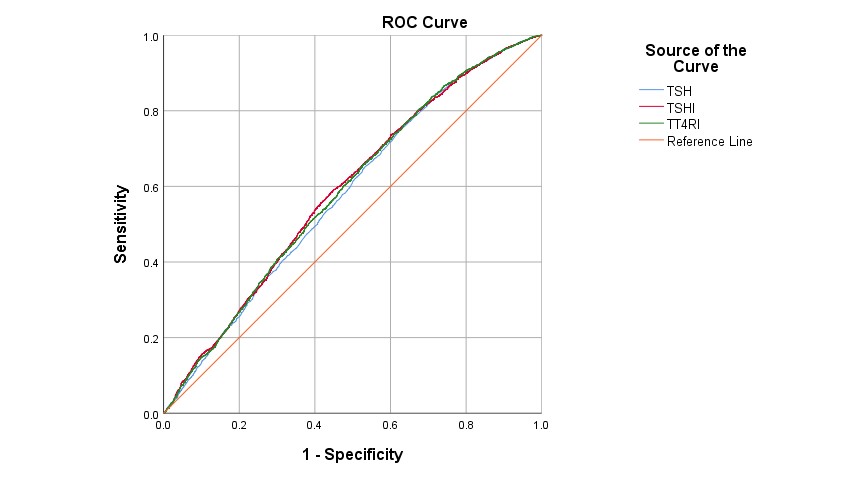


**Supplementary Figure S2.** ROC curve for thyroid parameters to distinguish differentiated thyroid cancer from benign disease.


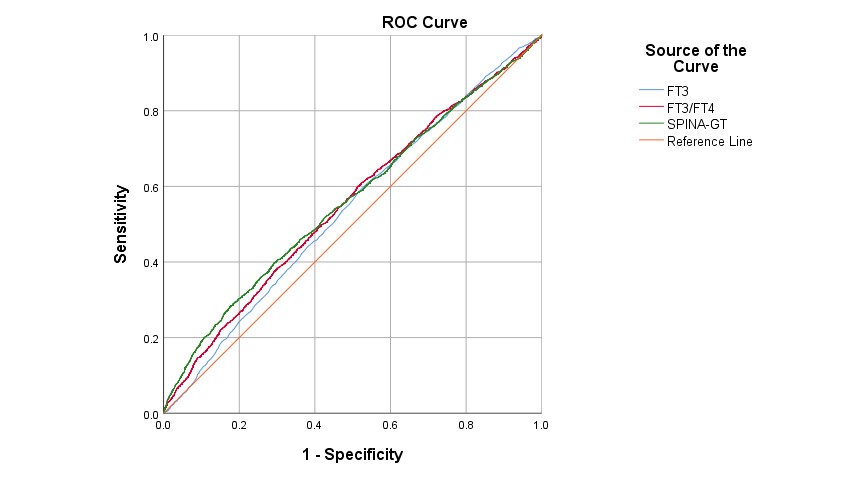


**Supplementary Figure S3.** ROC curve for thyroid parameters to distinguish benign disease from differentiated thyroid cancer.

# Supplementary Tables

**Supplementary Table S1.** Numbers of missing data for variables

|  | **Benign nodule group** | **Differentiated thyroid cancer group** | **Total** |
| --- | --- | --- | --- |
| BMI | 4 | 3 | 7 |
| Hashimoto thyroiditis confirmed by pathology | 4 | 11 | 15 |
| TgAb | 217 | 341 | 558 |
| TPOAb | 296 | 556 | 852 |
| Nodule size | 647 | 1218 | 1865 |

**Supplementary Table S2.** Association of thyroid parameters (quartiles) with differentiated thyroid cancer in total subjects

|  | **Crude** |  | **Model 1** |  | **Model 2** |  |
| --- | --- | --- | --- | --- | --- | --- |
|  | **OR (95%CI)** | ***P*** | **OR (95%CI)** | ***P*** | **OR (95%CI)** | ***P*** |
| FT3 |  |  |  |  |  |  |
| Q1 | 1.39 (1.21-1.59) | <0.001 | 1.84 (1.58-2.14) | <0.001 | 1.84 (1.58-2.15) | <0.001 |
| Q2 | 1.28 (1.12-1.47) | <0.001 | 1.59 (1.37-1.84) | <0.001 | 1.61 (1.39-1.87) | <0.001 |
| Q3 | 1.08 (0.94-1.24) | 0.261 | 1.23 (1.07-1.41) | 0.004 | 1.24 (1.08-1.43) | 0.003 |
| Q4 | 1 |  | 1 |  | 1 |  |
| *P* trend | <0.001 |  | <0.001 |  | <0.001 |  |
| FT4 |  |  |  |  |  |  |
| Q1 | 1 |  | 1 |  | 1 |  |
| Q2 | 1.22 (1.07-1.40) | 0.004 | 1.14 (0.99-1.31) | 0.069 | 1.15 (1.00-1.32) | 0.050 |
| Q3 | 1.31 (1.15-1.51) | <0.001 | 1.23 (1.07-1.42) | 0.004 | 1.26 (1.09-1.46) | 0.001 |
| Q4 | 1.30 (1.13-1.49) | <0.001 | 1.17 (1.01-1.34) | 0.035 | 1.17 (1.02-1.35) | 0.031 |
| *P* trend | <0.001 |  | 0.022 |  | 0.018 |  |
| TSH |  |  |  |  |  |  |
| Q1 | 1 |  | 1 |  | 1 |  |
| Q2 | 1.66 (1.45-1.89) | <0.001 | 1.60 (1.40-1.84) | <0.001 | 1.58 (1.38-1.81) | <0.001 |
| Q3 | 1.86 (1.62-2.13) | <0.001 | 1.81 (1.57-2.08) | <0.001 | 1.78 (1.55-2.05) | <0.001 |
| Q4 | 1.96 (1.71-2.25) | <0.001 | 1.95 (1.69-2.25) | <0.001 | 1.81 (1.57-2.09) | <0.001 |
| *P* trend | <0.001 |  | <0.001 |  | <0.001 |  |
| TSHI |  |  |  |  |  |  |
| Q1 | 1 |  | 1 |  | 1 |  |
| Q2 | 1.36 (1.19-1.55) | <0.001 | 1.51 (1.32-1.72) | <0.001 | 1.49 (1.30-1.71) | <0.001 |
| Q3 | 1.90 (1.66-2.18) | <0.001 | 2.02 (1.76-2.33) | <0.001 | 1.99 (1.72-2.29) | <0.001 |
| Q4 | 2.06 (1.79-2.37) | <0.001 | 2.02 (1.75-2.33) | <0.001 | 1.90 (1.64-2.19) | <0.001 |
| *P* trend | <0.001 |  | <0.001 |  | <0.001 |  |
| TT4RI |  |  |  |  |  |  |
| Q1 | 1 |  | 1 |  | 1 |  |
| Q2 | 1.61 (1.41-1.83) | <0.001 | 1.59 (1.39-1.82) | <0.001 | 1.56 (1.36-1.79) | <0.001 |
| Q3 | 1.92 (1.68-2.20) | <0.001 | 1.86 (1.62-2.14) | <0.001 | 1.83 (1.59-2.11) | <0.001 |
| Q4 | 2.10 (1.83-2.41) | <0.001 | 2.04 (1.77-2.35) | <0.001 | 1.90 (1.65-2.20) | <0.001 |
| *P* trend | <0.001 |  | <0.001 |  | <0.001 |  |
| FT3/FT4 | |  |  |  |  |  |
| Q1 | 1.67 (1.45-1.92) | <0.001 | 1.75 (1.52-2.02) | <0.001 | 1.78 (1.54-2.05) | <0.001 |
| Q2 | 1.39 (1.22-1.60) | <0.001 | 1.39 (1.21-1.60) | <0.001 | 1.42 (1.23-1.63) | <0.001 |
| Q3 | 1.24 (1.08-1.41) | 0.002 | 1.23 (1.07-1.40) | 0.004 | 1.25 (1.09-1.44) | 0.001 |
| Q4 | 1 |  | 1 |  | 1 |  |
| *P* trend | <0.001 |  | <0.001 |  | <0.001 |  |
| SPINA-GT | |  |  |  |  |  |
| Q1 | 1.73 (1.51-1.98) | <0.001 | 1.83 (1.59-2.11) | <0.001 | 1.73 (1.49-1.99) | <0.001 |
| Q2 | 1.61 (1.41-1.85) | <0.001 | 1.59 (1.39-1.83) | <0.001 | 1.55 (1.34-1.78) | <0.001 |
| Q3 | 1.57 (1.37-1.79) | <0.001 | 1.52 (1.33-1.75) | <0.001 | 1.53 (1.33-1.76) | <0.001 |
| Q4 | 1 |  | 1 |  | 1 |  |
| *P* trend | <0.001 |  | <0.001 |  | <0.001 |  |

The ORs (95% CI) of quartiles of thyroid parameters for differentiated thyroid cancer in different logistic regression models are shown.

Model 1: Adjusted for age and sex;

Model 2: Adjusted for age, sex, BMI, family history of thyroid cancer and Hashimoto thyroiditis confirmed by pathology.

Abbreviations: FT3, free triiodothyronine; FT4, free thyroxine; TSH, thyroid stimulating hormone; TSHI, TSH index; TT4RI, thyrotroph thyroxine resistance index; FT3/FT4, FT3/FT4 ratio; SPINA-GT, the thyroid’s secretory capacity.

**Supplementary Table S3.** Specific pathological types of benign disease

| **Type** | **No. of patients (%)** |
| --- | --- |
| Nodular goiter | 1422(68.76%) |
| Follicular adenoma | 461(22.29%) |
| Nodular goiter coexisting with follicular adenoma | 77(3.72%) |
| Hashimoto thyroiditis | 44(2.13%) |
| Subacute thyroiditis | 14(0.68%) |
| Other benign diseases | 50(2.42%) |

**Supplementary Table S4.** Thyroid parameters in euthyroid subjects with or without pathologically confirmed Hashimoto thyroiditis (HT)

| **Variables** | **Non-HT (n = 8171)** | **HT (n = 1329)** | ***P*** |
| --- | --- | --- | --- |
| FT3, pmol/L | 4.72±0.52 | 4.59±0.50 | <0.001 |
| FT4, pmol/L | 15.19±2.16 | 14.96±2.11 | <0.001 |
| TSH, mU/L | 1.85 (1.25, 2.68) | 2.27 (1.55, 3.29) | <0.001 |
| TSHI | 2.63±0.58 | 2.78±0.58 | <0.001 |
| TT4RI | 27.96 (18.93, 40.05) | 33.54 (22.69, 47.82) | <0.001 |
| FT3/FT4 | 0.313 (0.285, 0.344) | 0.309 (0.281, 0.342) | 0.004 |
| SPINA-GT, pmol/s | 2.86 (2.26, 3.76) | 2.50 (2.04, 3.18) | <0.001 |
| TgAb, IU/ml | 15.00 (15.00, 25.40) | 145.70 (61.30, 278.55) | <0.001 |
| TPOAb, IU/ml | 28.00(27.00, 39.30) | 109.20 (31.30, 1072.10) | <0.001 |

Data are expressed as mean ± standard deviation for normally distributed variables and median (interquartile range) for skewed distributed variables.

Abbreviations: TSHI, TSH index; TT4RI, thyrotroph thyroxine resistance index; FT3/FT4, FT3/FT4 ratio; SPINA-GT, the thyroid’s secretory capacity; TgAb, serum thyroglobulin antibody; TPOAb, serum thyroid peroxidase antibody.
